# Update of the pediatric hypotension graphic adjusted for gender and height percentiles: systolic blood pressure for boys, 1 to 17 years old

**DOI:** 10.1186/cc12637

**Published:** 2013-06-19

**Authors:** HH Shieh, ER Barreira, A Bousso, AC Ventura, EJ Troster

**Affiliations:** 1 University Hospital of Universidade de São Paulo, Vila Iara, São Paulo, SP, Brazil

## Introduction

According to the National Heart, Lung, and Blood Institute of the National Institute of Health, hypotension refers to an abnormally low blood pressure (BP). In childhood, hypotension can be determined according to two different definitions: BP below the 5th percentile or below two standard deviations (SDs) of the mean for age and gender [1]. A graphic representation of pediatric hypotension was published in 1977 [2], and no updates have been published since then. The objective of this study was to update the graphic representation of pediatric hypotension.

## Methods

We used a computerized calculation method to develop high-resolution graphics containing curves with 5,841 points each, to depict the main percentiles associated with low BP for boys from 1 to 17 years old in the 50th percentile of height. Each point represents the calculation of the polynomial equation that includes the statistical processing of the last Report on Blood Pressure [3]. We also analyzed the effect of height on BP in the 5th to 95th percentile range. Statistical functions generated by computerized program were used.

## Results

**Figure 1:**
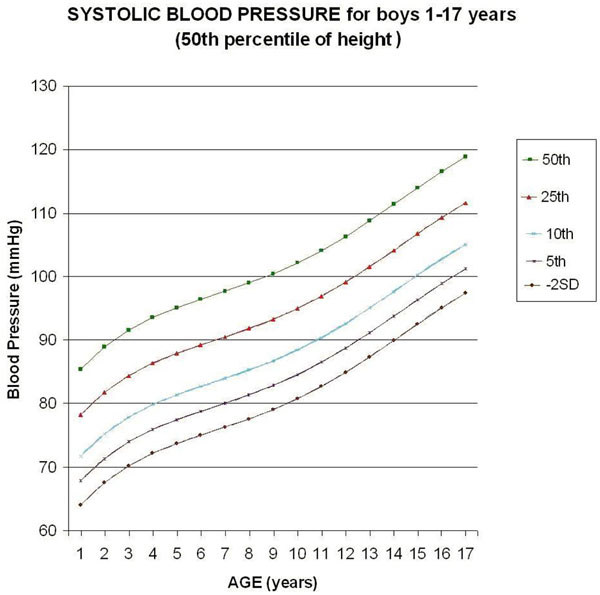
**Update of chart for systolic blood pressure (SBP) based on the last Report on Blood Pressure in 2004 **[3]**, for boys 1 to 17 years old (50th percentile of height)**. Considering a tolerance of 1 mmHg, the curve of adjustment for height of the SBP for boys does not need any correction in the in the 36.5th to 64.5th percentile of height.

Five monotonic curves of systolic BP for boys representing the 50th, 25th, 10th, 5th, and 2.275th (-2SD) percentiles were built (Figure 1). In relation to the table published by Haque and Zaritzsky [4], the Bland-Altman analysis of the published male systolic BP compared with actual update shows a mean bias of -0.53, with limits of agreement of -2.01 to 0.96 mmHg, which confirms the use of approximation of the values in the published table [4]. Considering a tolerance of 1 mmHg, the monotonic curve of adjustment for height of the systolic BP for boys does not need any correction in the 36.5th to 64.5th percentile of height, but needs maximal correction for the 95th percentile of height (+3.8 mmHg correction).

## Conclusion

The adjustment of systolic BP for height is of little significance, and the updated graphic can be used to diagnose low systolic BP for boys. Clinical studies are necessary to determine the systolic BP percentile that better represents clinically significant hypotension.

## References

[B1] Shieh HH Gilio AE Barreira ER Troster EJ Ventura AMC Goes PF Souza DC Sinimbu Filho JM Bousso A Pediatric hypotension: quantification of the differences between the two current definitions Intensive Care Med 2012 17 Suppl 1 S0662. doi: 10.1007/s00134-012-2683-0

[B2] Blumenthal S Report of the Task Force on Blood Pressure Control in Children Pediatrics 1977 17 Suppl 797 859728

[B3] National High Blood Pressure Education Program Working Group on High Blood Pressure in Children and Adolescents: the fourth report on the diagnosis, evaluation, and treatment of high blood pressure in children and adolescents Pediatrics 2004 17 Suppl 4th Report 555 576 15286277

[B4] Haque IU Zaritsky AL Analysis of the evidence for the lower limit of systolic and mean arterial pressure in children Pediatr Crit Care Med 2008 17 138 144 10.1097/01.PCC.0000257039.32593.DC 17273118

